# Bone Mineral Density in Women with Functional Hypothalamic Amenorrhea: A Systematic Review and Meta-Analysis

**DOI:** 10.3390/medsci14060615

**Published:** 2026-09-29

**Authors:** Zhanat Sultanova, Saule Issenova, Yelena Dissyukeyeva, Saule Nukusheva, Aliya Aimbetova, Kulman Nyssanbayeva, Rassul Dyussenov, Fatima Kassymbekova

**Affiliations:** 1 Department of Obstetrics and Gynecology, Asfendiyarov Kazakh National Medical University, Almaty 050000, Kazakhstan; sultanova.zh@kaznmu.kz (Z.S.); isenova.s@kaznmu.kz (S.I.); 2 Department of Obstetrics and Gynecology No. 2, Astana Medical University, Astana 010000, Kazakhstan; 3 Department of Health Policy and Management, Asfendiyarov Kazakh National Medical University, Almaty 050000, Kazakhstan; 4 Board for Clinical Work, Scientific Center of Obstetrics, Gynecology and Perinatology, Almaty 050020, Kazakhstan; 5 Department of Propedeutics of Internal Diseases, Asfendiyarov Kazakh National Medical University, Almaty 050000, Kazakhstan; nysanbayeva.k@kaznmu.kz; 6 Department of Surgical Diseases, Kazakhstani Medical University “KSPH”, Almaty 050060, Kazakhstan

**Keywords:** functional hypothalamic amenorrhea, bone mineral density, anorexia nervosa, dual-energy X-ray absorptiometry, osteoporosis, systematic review, meta-analysis

## Abstract

This study aims to quantitatively evaluate bone mineral density (BMD) in women with functional hypothalamic amenorrhea (FHA), determine differences across skeletal sites, and summarise current evidence on the diagnosis and management of impaired bone health in this population. **Methods**: A systematic search of comparative studies evaluating BMD by dual-energy X-ray absorptiometry (DXA) in women with FHA or anorexia nervosa (AN) and healthy eumenorrhoeic controls was performed. Eleven studies met the eligibility criteria, five of which were included in the meta-analysis. The quantitative synthesis comprised 14 group comparisons, with lumbar spine and hip BMD analysed separately. Effect sizes were pooled using Hedges’ g with 95% confidence intervals (CI) under a random-effects model. Sensitivity analyses using REML estimation with Hartung–Knapp adjustment and leave-one-out analyses were performed to assess the robustness of the pooled estimates. **Results**: The narrative synthesis of 11 studies generally showed lower BMD in women with FHA or AN compared with healthy controls. In the meta-analysis, among women with FHA, hip BMD was significantly lower than in healthy controls (Hedges’ g = −0.60; 95% CI −1.13 to −0.06; *p* = 0.030; I^2^ = 63%), while a moderate reduction was observed at the lumbar spine (Hedges’ g = −0.68; 95% CI −1.48 to 0.13; *p* = 0.101; I^2^ = 83%). In women with AN, substantial reductions were observed at both the lumbar spine (Hedges’ g = −1.66; 95% CI −2.28 to −1.04; *p* < 0.001; I^2^ = 71%) and the hip (Hedges’ g = −1.37; 95% CI −2.33 to −0.42; *p* = 0.005; I^2^ = 89%). The lumbar spine reduction in AN remained statistically significant across sensitivity and leave-one-out analyses and was the most robust finding, whereas the statistical significance of hip BMD estimates in both FHA and AN and of lumbar spine BMD in FHA was sensitive to the analytical approach or exclusion of individual studies. The systematic review demonstrated that DXA with interpretation based on Z-scores remains the preferred method for skeletal assessment in premenopausal women with FHA. Bone health assessment is recommended after ≥6 months of amenorrhea or earlier in women with severe energy deficiency or a history of fractures. Restoration of energy availability, weight gain, and recovery of menstrual function remain the cornerstone of treatment. When amenorrhea persists and low BMD is confirmed, physiological transdermal 17β-estradiol combined with cyclic progesterone is the preferred hormonal therapy, whereas combined oral contraceptives, bisphosphonates, and denosumab are not recommended for routine management. **Conclusions**: Functional hypothalamic amenorrhea is associated with reduced BMD, with substantially greater skeletal deficits observed in the AN subgroup. These findings support early assessment of bone health and timely correction of low energy availability in women with FHA. The considerable between-study heterogeneity and sensitivity of some pooled estimates highlight the need for larger prospective studies using standardized diagnostic criteria and uniform skeletal outcome measures.

## 1. Introduction

Functional hypothalamic amenorrhea (FHA) is a reversible form of hypogonadotropic hypogonadism caused by functional suppression of pulsatile hypothalamic gonadotropin-releasing hormone (GnRH) secretion in the absence of structural abnormalities of the central nervous system, pituitary gland, ovaries, or uterus. It is a diagnosis of exclusion and most commonly develops in response to energy deficiency, excessive physical exercise, restrictive eating behaviors, weight loss, chronic psychological stress, or a combination of these factors [1,2]. FHA is one of the leading causes of secondary amenorrhea (accounting for roughly 20% to 35% of secondary amenorrhea cases) in women of reproductive age [3]. Anorexia nervosa (AN) is a well-recognized cause of functional hypothalamic amenorrhea and is associated with severe and persistent low energy availability, making it an important clinical model for investigating the effects of chronic energy deficiency on bone health [4].

Although menstrual dysfunction associated with FHA is potentially reversible, its clinical significance extends far beyond reproductive health. Among its long-term systemic consequences, impaired skeletal health is considered one of the most clinically important because it develops during the critical period of peak bone mass acquisition. Approximately 90% of peak bone mass is attained by the end of the second decade of life, whereas skeletal mineralization continues until approximately 25–30 years of age [1,5]. While hypoestrogenism accelerates bone resorption and suppresses bone formation, skeletal deterioration in FHA cannot be explained by estrogen deficiency alone. Low energy availability is accompanied by reduced circulating concentrations of insulin-like growth factor-1 (IGF-1) and leptin, relative hypercortisolemia, alterations in thyroid hormone metabolism, and inadequate nutrient intake, all of which adversely affect bone mineral density (BMD), bone microarchitecture, and mechanical strength [6,7]. These alterations are particularly detrimental during adolescence and early adulthood, when peak bone mass is still being established. Consequently, prolonged amenorrhea during this period may simultaneously impair bone accrual and accelerate bone loss, resulting in persistent deficits in skeletal reserve [8].

Observational studies have consistently demonstrated reduced BMD in women with hypothalamic amenorrhea, with the lumbar spine being the most vulnerable skeletal site. In a prospective study by Biller et al., spinal BMD was below the age-adjusted mean in 20 of 24 women with hypothalamic amenorrhea, while eight participants had values at least two standard deviations below the reference mean [9]. More recent studies have confirmed significantly lower BMD at the lumbar spine, femoral neck, and whole body in women with FHA compared with healthy eumenorrheic controls [10,11].

The severity of skeletal impairment appears to depend on several factors, including the duration of amenorrhea, age at onset, body mass index, energy availability, and exercise characteristics. Dual-energy X-ray absorptiometry (DXA) remains the reference standard for assessing bone health in this population, with results in adolescents and premenopausal women interpreted primarily using Z-scores in conjunction with the clinical context.

Despite the growing body of literature, the available evidence remains clinically and methodologically heterogeneous, and the magnitude of bone loss across different skeletal sites has not been clearly established. Therefore, the aim of this systematic review and meta-analysis was to quantitatively evaluate reductions in BMD in women with FHA compared with healthy eumenorrheic controls, determine site-specific differences in skeletal involvement, and summarize current evidence regarding the diagnosis and clinical management of impaired bone health in women with FHA. Because anorexia nervosa is a common cause of FHA and represents a clinical model of severe chronic low energy availability, studies involving women with AN were also evaluated separately to characterize the spectrum of skeletal impairment associated with this condition.

## 2. Materials and Methods

### 2.1. Study Design

This systematic review and meta-analysis was conducted and reported in accordance with the Preferred Reporting Items for Systematic Reviews and Meta-Analyses (PRISMA 2020) statement. The PRISMA 2020 Checklist is provided as Table S1. This study was conducted as a systematic review with a meta-analysis of comparative studies. The systematic review summarized current evidence on the pathophysiology, diagnosis, and management of impaired bone health in women with FHA, whereas the meta-analysis quantitatively evaluated reductions in BMD in women with FHA compared with healthy controls. Studies of women with FHA were analyzed separately from studies specifically enrolling women with AN. In the studies contributing to the FHA analyses, AN was either explicitly excluded or the study population was selected to represent non-AN causes of hypothalamic amenorrhea.

### 2.2. Search Strategy and Eligibility Criteria

A systematic literature search was conducted in Google Scholar, MEDLINE/PubMed, and Web of Science and included English-language publications published between 1990 and 2026. The literature search was last updated on 26 May 2026. The search strategy combined the following keywords: functional hypothalamic amenorrhea, hypothalamic amenorrhea, exercise-associated amenorrhea, amenorrheic athletes, anorexia nervosa, bone mineral density, DXA, lumbar spine, femoral neck, total hip, and eumenorrheic controls. Studies involving women with AN were included because AN represents one of the principal etiological forms of FHA. Following peer review, the literature search was expanded to include Scopus using the same search terms and eligibility criteria as in the original search. The supplementary Scopus search was conducted on 28 August 2026.

Studies were considered eligible if they met the following criteria: (1) primary comparative studies including women with FHA; (2) quantitative BMD measurements obtained by DXA; (3) a healthy eumenorrheic control group; and (4) publication in the English language. Studies were excluded if they (1) did not report quantitative BMD measurements or lacked a healthy control group, (2) investigated forms of amenorrhea other than FHA, or (3) were conference abstracts, case reports, letters, commentaries, or editorials. Articles with insufficient data for comparison with the control group, as well as studies for which the full text was unavailable, were also excluded.

The protocol for this systematic review and meta-analysis was not prospectively registered in the International Prospective Register of Systematic Reviews (PROSPERO), which limits transparency regarding the prespecification of the review methods. This should be considered when interpreting the findings. The search was restricted to English-language publications, which may have resulted in the omission of relevant studies and represents a limitation of the present review.

### 2.3. Study Selection and Data Extraction

**Figure 1:**
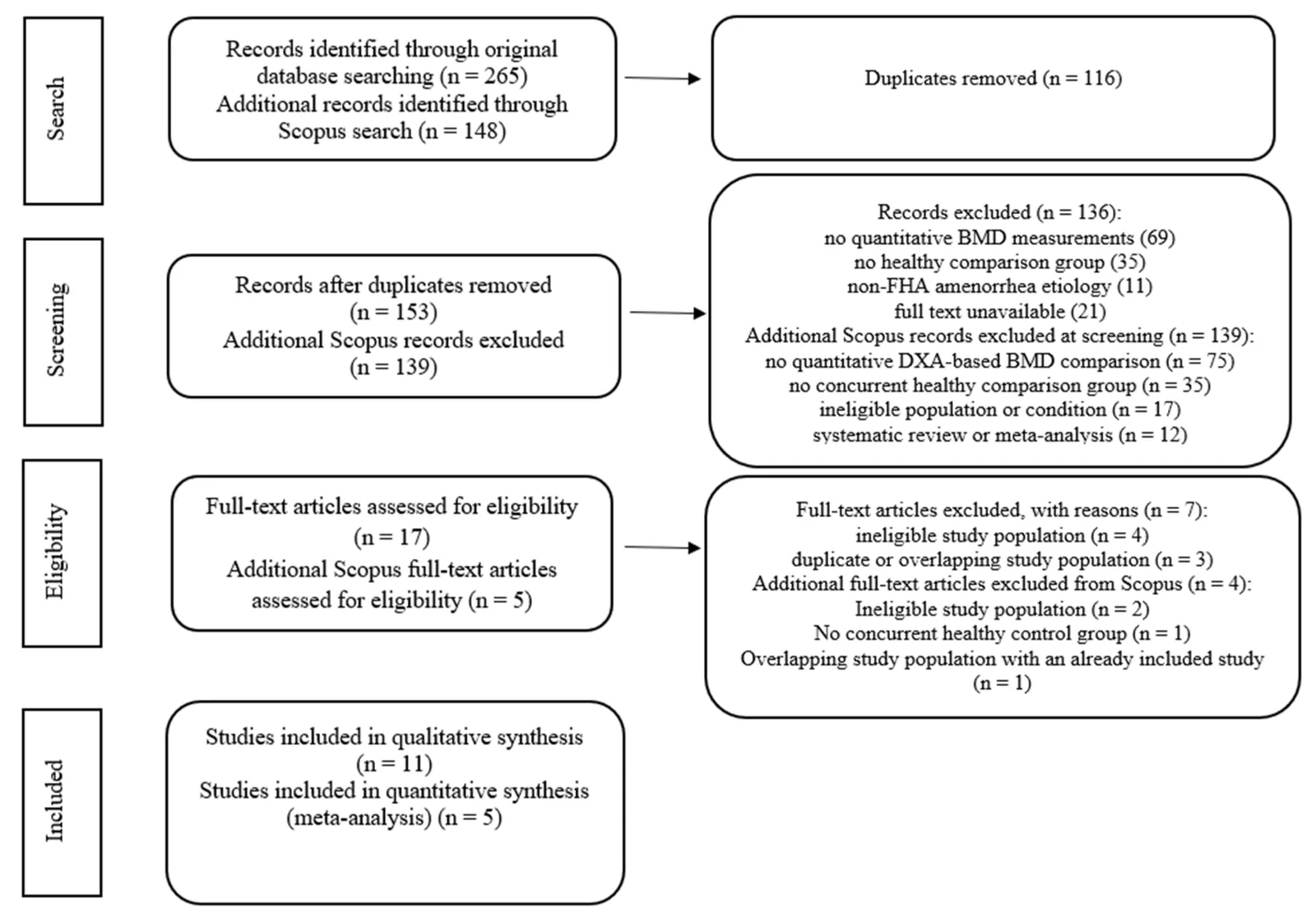
Preferred Reporting Items for Systematic Reviews and Meta-Analyses (PRISMA) 2020 flow diagram of the study selection process.

The initial search identified 265 records. After removal of duplicate records and exclusion of studies that did not meet the eligibility criteria, 10 publications were included in the systematic review. The supplementary Scopus search identified 148 records. Following title and abstract screening, five articles were assessed for eligibility in full text. Four were excluded because of an ineligible study population (*n* = 2), absence of a concurrent healthy control group (*n* = 1), or an overlapping study population with an already included study (*n* = 1). One additional study (Safwan et al., 2026) [12] met the eligibility criteria and was included in the qualitative synthesis. This study was not included in the meta-analysis because BMD data were reported as median [IQR], which did not permit direct calculation of the effect estimates used in the quantitative synthesis. Combining both search strategies, a total of 11 studies were included in the systematic review, of which five provided data suitable for the meta-analysis and six were included in the narrative synthesis only. The study selection process is illustrated in Figure 1. Titles and abstracts identified through the database search were screened for eligibility, followed by full-text assessment of potentially relevant articles according to the predefined inclusion and exclusion criteria. Studies meeting all eligibility criteria were included in the qualitative synthesis, and those providing sufficient quantitative data were included in the meta-analysis.

Data extracted from each eligible study included study design, country, participant characteristics, sample size, age, diagnostic criteria for FHA, DXA system, skeletal sites evaluated, BMD outcomes, and the principal findings. These data were summarized in a standardized evidence table and used for the qualitative synthesis and quantitative meta-analysis where appropriate.

### 2.4. Meta-Analysis

Studies reporting mean BMD values with corresponding measures of variability that permitted calculation of standard deviations for the lumbar spine and hip separately in the FHA and control groups were eligible for inclusion in the meta-analysis. Five studies, comprising a total of 14 pairwise group comparisons, were included. Separate meta-analyses were performed for lumbar spine and hip BMD in women with FHA and in those with AN.

Women with AN were analysed separately because, although anorexia nervosa represents one of the etiological forms of FHA, it is a distinct clinical entity characterised by a more severe and qualitatively different pattern of energy deficiency.

Given the relatively small sample sizes of the included studies, between-group differences in BMD were expressed as Hedges’ *g*, a bias-corrected standardized mean difference. Hedges’ *g* was calculated as

*g* = *J* × [(*M*_1_ − *M*_2_)/*SD*_pooled_],

where *J* = 1 − 3/(4df − 1) is the small-sample correction factor and df = n1 + n2 − 2.

For the two studies that reported the standard error of the mean (*SEM*) rather than the standard deviation (*SD*), *SD* was calculated as

*SD* = *SEM* × √*n*,

where n represents the corresponding group sample size. All BMD values across the five studies included in the meta-analysis were reported in the same unit (g/cm^2^). Lumbar spine and hip BMD were analyzed as separate outcomes, with independent pooled estimates calculated for each skeletal site.

The primary meta-analysis was performed using the DerSimonian–Laird random-effects model, consistent with the original analytical approach, and statistical heterogeneity was quantified using the I^2^ statistic. Results are presented as pooled Hedges’ g values with corresponding 95% confidence intervals (CIs). Assessment of publication bias was not performed because fewer than ten studies were available for quantitative synthesis, which limits the reliability of funnel plot-based methods. Given the small number of studies and the observed between-study heterogeneity, an additional sensitivity analysis was performed using restricted maximum likelihood (REML) estimation with the Hartung–Knapp adjustment for 95% confidence intervals to assess the robustness of the pooled estimates to the choice of random-effects method. In addition, leave-one-out sensitivity analyses were performed by sequentially excluding each study and recalculating the pooled estimate using the DerSimonian–Laird random-effects model to assess the influence of individual studies on the overall findings.

The methodological quality of the studies included in the meta-analysis was assessed using the Newcastle–Ottawa Scale (NOS) for case–control studies. The NOS evaluates three domains: selection of study groups, comparability of groups, and ascertainment of exposure, with a maximum score of 9 points. Detailed item-level assessments for each study are provided in Supplementary Table S2.

All meta-analytic calculations, including the primary DerSimonian–Laird models, REML analyses with the Hartung–Knapp adjustment, and leave-one-out sensitivity analyses, were performed in R version 4.3.3 using the metafor package (version 2026.08.2). Statistical significance was defined as a two-sided *p* value < 0.05.

## 3. Results

### 3.1. Study Characteristics and Descriptive Analysis

Eleven comparative studies comprising a total of 822 women with functional hypothalamic amenorrhea (FHA) or anorexia nervosa (AN) and 449 healthy controls were included in the systematic review (Table 1). Most studies employed a cross-sectional comparative design, whereas one study had a retrospective design [13]. The study populations included women with FHA, AN, and athletes with amenorrhea or oligomenorrhea, together with healthy eumenorrheic controls. Participants ranged in age from adolescence to the reproductive years. In all studies, BMD was assessed by dual-energy X-ray absorptiometry (DXA). In addition, Ackerman et al. (2011) [14] evaluated bone microarchitecture using high-resolution peripheral quantitative computed tomography (HR-pQCT).

Across all included studies, amenorrhea was consistently associated with lower BMD, with the greatest skeletal deficits observed in women with AN [13,15,16]. The direction of the association remained consistent regardless of country, year of publication, or DXA system used. Notably, Safwan et al. (2026) additionally compared women with FHA with recently menopausal women and found no statistically significant difference in lumbar spine BMD between the groups (*p* = 0.07) [12]. Among athletes, bone loss predominantly affected the lumbar spine, whereas hip BMD was generally preserved. However, HR-pQCT demonstrated impaired trabecular bone microarchitecture even in participants with normal BMD values on DXA [14,17].

Factors associated with reduced BMD varied according to both skeletal site and the underlying etiology of amenorrhea. Lower BMD was associated with reduced lean body mass, lower circulating insulin-like growth factor-1 (IGF-1) concentrations, longer duration of amenorrhea, elevated cortisol levels, and greater severity of anxiety and depressive symptoms [13,15,18]. A history of amenorrhea during adolescence increased the risk of low adult BMD by more than seven fold (odds ratio, 7.11), independently of current menstrual status [19].

Among athletes, skeletal changes were site-specific. Ackerman et al. (2011) [14] reported that athletes with amenorrhea had significantly lower lumbar spine BMD than both eumenorrheic athletes and non-athletic controls, whereas no significant differences were observed in hip or femoral neck BMD.

**Table 1:** Characteristics of Studies Included in the Systematic Review.

Study	Country, Study Design	Study Population	Age (Years)	DXA System/Skeletal Sites	Data Format	Main Findings
Notaristefano et al., 2022 [20]	Italy, cross-sectional comparative	FHA without AN (*n* = 32); controls (*n* = 19)	Not reported separately (reproductive age)	Lunar; lumbar spine, total hip, femoral neck	Mean ± SD; included in meta-analysis	Total hip and femoral neck BMD were significantly lower in women with FHA. Absolute lumbar spine BMD was not significantly different, although lumbar spine Z-scores were significantly reduced.
Grinspoon et al., 1999 [15]	USA, three-arm cross-sectional comparative	FHA (*n* = 19); AN (*n* = 30); controls (*n* = 30)	24 ± 1 in all groups	Hologic 4500; lumbar spine, total hip, whole body	Mean ± SEM; included in meta-analysis	BMD was reduced in both amenorrheic groups, with the lowest values observed in AN. Lean body mass was the strongest independent predictor of BMD.
Lawson et al., 2009 [18]	USA, three-arm cross-sectional comparative	FHA (*n* = 13); AN (*n* = 18); controls (*n* = 21)	Mean 25–27	Hologic 4500; lumbar spine, lateral spine, total hip	Mean ± SEM; included in meta-analysis	Cortisol levels were inversely associated with BMD at all skeletal sites. Anxiety and depressive symptoms were also negatively associated with BMD.
Ackerman et al., 2011 [14]	USA, three-arm cross-sectional comparative	Amenorrheic athletes with FHA (*n* = 16); eumenorrheic athletes (*n* = 18); non-athletic controls (*n* = 15)	15–21	Hologic 4500 (DXA) + HR-pQCT; lumbar spine, hip, distal radius, distal tibia	Mean ± SD; included in meta-analysis	Amenorrheic athletes had lower lumbar spine BMD despite relatively preserved hip BMD. HR-pQCT demonstrated impaired trabecular microarchitecture independent of DXA findings.
Trombetti et al., 2013 [13]	Switzerland, retrospective comparative	AN (*n* = 66); controls (*n* = 66)	Median 20 (16–33)	Hologic; lumbar spine, total hip, femoral neck	Mean ± SD; included in meta-analysis	IGF-1 was the main independent predictor of hip BMD, whereas duration of amenorrhea independently predicted lumbar spine BMD.
Haenggi et al., 1994 [21]	Switzerland, cross-sectional comparative	Hypothalamic/ovarian amenorrhea (*n* = 21); controls (*n* = 123)	Not reported	Not reported; lumbar spine, femoral neck	Percentage of control mean; descriptive synthesis	Lumbar spine BMD was 85% and femoral neck BMD 92% of control values.
Kandemir et al., 2018 [16]	USA, three-arm cross-sectional comparative	AN (*n* = 269); oligoamenorrheic athletes (*n* = 104); controls (*n* = 95)	14–21.9	Not reported; lumbar spine, hip	Proportion of participants with Z-score < −1; descriptive synthesis	The prevalence of low BMD increased progressively from controls to oligoamenorrheic athletes and was highest in women with AN at both skeletal sites.
Nose-Ogura et al., 2020 [19]	Japan, cross-sectional comparative (elite athlete cohort)	Elite athletes (*n* = 210)	Approximately 20	Not reported; lumbar spine	Odds ratio; descriptive synthesis	Adolescent amenorrhea increased the risk of low adult BMD more than seven fold (OR 7.11), independent of current menstrual status.
Davies et al., 1990 [22]	United Kingdom, cross-sectional comparative	Mixed-etiology amenorrhea (*n* = 200); controls (*n* = 57)	16–40	Not reported; lumbar spine (L1–L4)	Mean ± SD; mixed etiology	Lumbar spine BMD was 15% lower than in controls (95% CI 12–18%): 0.89 ± 0.12 vs. 1.05 ± 0.09 g/cm^2^.
Drinkwater et al., 1984 [17]	USA, cross-sectional comparative	Amenorrheic athletes (*n* = 14); eumenorrheic athletes (*n* = 14)	Age not reported; groups were age-matched	Not reported; lumbar spine (L1–L4)	Mean values only; not included in meta-analysis	Lumbar spine BMD was lower in amenorrheic than in eumenorrheic athletes (1.12 vs. 1.30 g/cm^2^).
Safwan et al., 2026 [12]	USA, cross-sectional comparative	FHA (*n* = 20); eumenorrheic controls (*n* = 9); also compared to recently menopausal women (*n* = 12)	Median 27.8 [23.4, 34.1] vs. 29.6 [28.3, 32.4]	L1–4, L2–4, femoral neck, total femur, spine, total BMD (all by DXA, Lunar iDXA)	Median [IQR], not included in meta-analysis	Lumbar spine BMD significantly lower in FHA vs. controls (*p* = 0.04); FHA not significantly different from recently menopausal women (*p* = 0.07).

Abbreviations: AN, anorexia nervosa; BMD, bone mineral density; CI, confidence interval; DXA, dual-energy X-ray absorptiometry; FHA, functional hypothalamic amenorrhea; HR-pQCT, high-resolution peripheral quantitative computed tomography; IGF-1, insulin-like growth factor-1; IQR, interquartile range; OR, odds ratio; SD, standard deviation; SEM, standard error of the mean.

### 3.2. Meta-Analysis

Five studies comprising a total of 14 pairwise comparisons of lumbar spine and hip BMD were included in the meta-analysis. Separate analyses were performed according to the clinical subtype of amenorrhea and skeletal site. Overall, pooled effect estimates indicated lower BMD across all study groups compared with healthy controls, although statistical significance varied by clinical subgroup and skeletal site.

**Figure 2:**
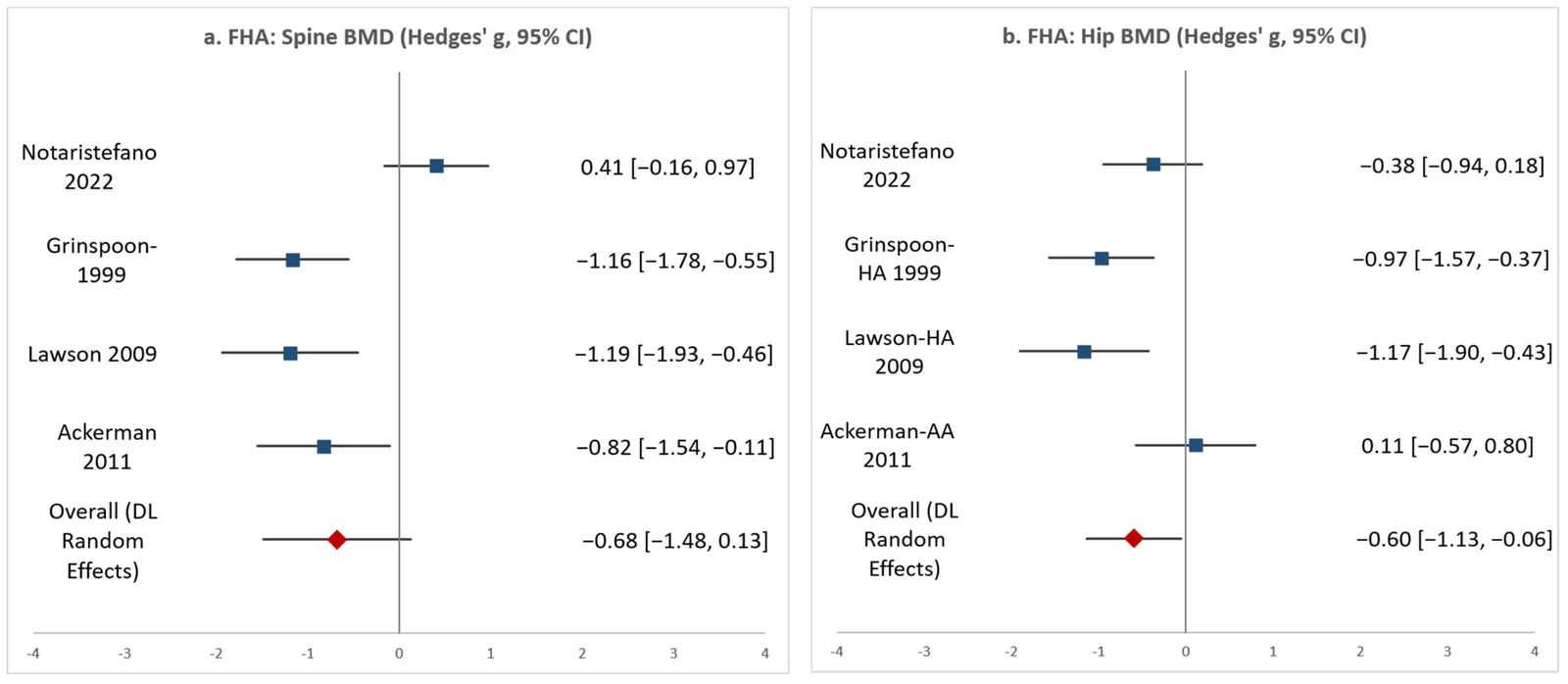
Forest plot of lumbar spine (**a**) and hip (**b**) bone mineral density (BMD) in women with functional hypothalamic amenorrhea (FHA) compared with healthy controls. Studies included in the meta-analysis: Notaristefano et al. [20], Grinspoon et al. [15], Lawson et al. [18], and Ackerman et al. [14]. CI, confidence interval.

Among women with FHA, the lumbar spine estimate indicated a moderate reduction in BMD (Hedges’ g = −0.68; 95% CI, −1.48 to 0.13; *p* = 0.101; I^2^ = 83%; Figure 2a). In contrast, hip BMD was significantly lower than in healthy controls, with a moderate effect size (Hedges’ g = −0.60; 95% CI, −1.13 to −0.06; *p* = 0.030; I^2^ = 63%; Figure 2b).

**Figure 3:**
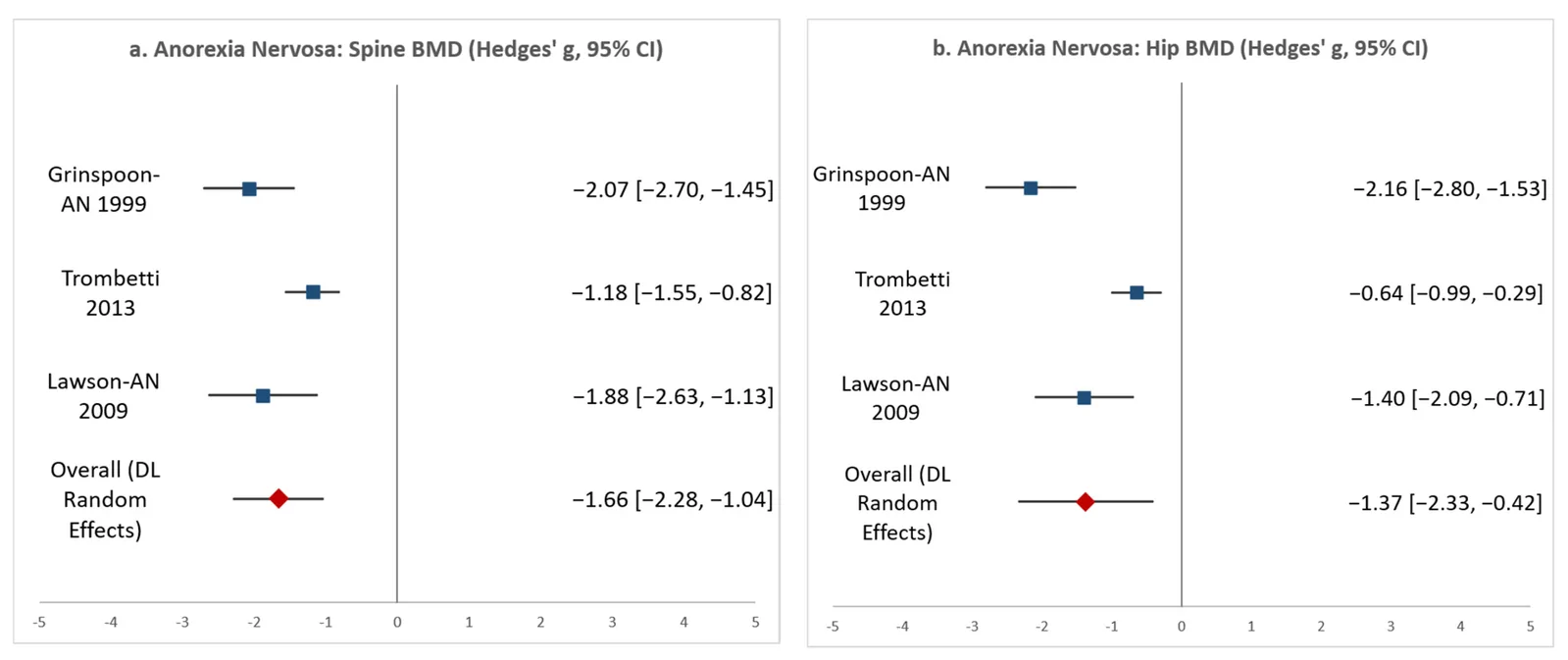
Forest plot of lumbar spine (**a**) and hip (**b**) bone mineral density (BMD) in women with anorexia nervosa (AN) compared with healthy controls. Studies included in the meta-analysis: Grinspoon et al. [15], Trombetti et al. [13], and Lawson et al. [18]. CI, confidence interval.

In women with AN, reductions in BMD were substantially greater. Large and statistically significant effect sizes were observed for both the lumbar spine (Hedges’ g = −1.66; 95% CI, −2.28 to −1.04; *p* < 0.001; I^2^ = 71%; Figure 3a) and the hip (Hedges’ g = −1.37; 95% CI, −2.33 to −0.42; *p* = 0.005; I^2^ = 89%; Figure 3b). Between-study heterogeneity was moderate to high across all analyses and was greatest for hip BMD in women with AN. The pooled effect estimates are presented in Table 2. Four of the five studies were rated as having good methodological quality according to the Newcastle–Ottawa Scale, with scores of 7–8 points, whereas Trombetti et al. [13] received a score of 6 points. The lower score for Trombetti et al. primarily reflected limitations in control selection and comparability between groups. Detailed item-level assessments are presented in Supplementary Table S2.

#### Sensitivity Analysis

Sensitivity analysis using REML estimation with the Hartung–Knapp adjustment produced pooled effect estimates that were similar in magnitude and direction to those obtained with the primary DerSimonian–Laird model, but with wider 95% CIs (Supplementary Table S3). The reduction in lumbar spine BMD in the AN subgroup remained statistically significant (g = −1.65, 95% CI −2.87 to −0.43; *p* = 0.028), whereas the reductions in hip BMD in the FHA (g = −0.60, 95% CI −1.50 to 0.31; *p* = 0.127) and AN (g = −1.37, 95% CI −3.29 to 0.55; *p* = 0.092) subgroups were no longer statistically significant. The lumbar spine estimate in the FHA subgroup remained non-significant. Overall, the magnitude and direction of the pooled effects were stable, although statistical significance for some comparisons was sensitive to the method used to account for uncertainty in the presence of a small number of studies.

Leave-one-out analyses were performed to assess the influence of individual studies on the pooled estimates (Supplementary Table S4). For lumbar spine BMD in the FHA subgroup, exclusion of Notaristefano et al. changed the pooled estimate from non-significant to significant (Hedges’ g = −1.07; 95% CI, −1.47 to −0.67) and reduced heterogeneity from I^2^ = 83% to 0%, whereas exclusion of the other studies did not alter statistical significance. For hip BMD in the FHA subgroup, the pooled estimate became non-significant after exclusion of Notaristefano et al., Grinspoon et al., or Lawson et al., but remained significant after exclusion of Ackerman et al. (Hedges’ g = −0.80; 95% CI, −1.27 to −0.33; I^2^ = 39%). For lumbar spine BMD in the AN subgroup, the pooled estimate remained statistically significant after exclusion of each individual study, with Hedges’ g ranging from −1.45 to −1.99 and heterogeneity ranging from 0% to 82%. For hip BMD in the AN subgroup, the pooled estimate remained significant after exclusion of Grinspoon et al. or Trombetti et al., but became non-significant after exclusion of Lawson et al. (Hedges’ g = −1.38; 95% CI, −2.87 to 0.11; I^2^ = 94%).

Overall, the statistical significance of the hip BMD estimates in both FHA and AN and of the lumbar spine estimate in FHA was sensitive to the analytical approach or exclusion of individual studies, whereas the lumbar spine reduction in AN remained statistically significant across sensitivity and leave-one-out analyses and represented the most robust pooled finding.

## 4. Overview of Bone Health Assessment and Management in Functional Hypothalamic Amenorrhea

This section provides a brief narrative overview of current guidelines and secondary evidence on bone health management in FHA and is intended to provide clinical context for the findings of the systematic review and meta-analysis.

### 4.1. Bone Mineral Density Assessment and Interpretation

The current Endocrine Society Clinical Practice Guideline recommends baseline BMD assessment by DXA in women with amenorrhea lasting six months or longer. Earlier evaluation is recommended in the presence of severe nutritional deficiency, eating disorders, delayed menarche, or a history of fragility fractures [1,23]. DXA assessment should include the lumbar spine and proximal femur, with whole-body measurements additionally recommended in adolescents [2].

In premenopausal women, DXA findings should be interpreted primarily using Z-scores rather than T-scores. A Z-score of ≤−2.0 is considered below the expected range for age, although findings should be interpreted in conjunction with individual clinical risk factors [24]. In contemporary clinical practice, DXA provides not only BMD measurements but also trabecular bone score (TBS), which complements BMD by assessing bone microarchitecture and improving fracture risk evaluation. TBS and high-resolution imaging techniques remain research tools and are not currently incorporated into routine clinical practice [2].

### 4.2. Management and Follow-Up

Restoration of energy availability is the cornerstone of management in FHA and should focus on adequate caloric intake, reduction in excessive exercise when appropriate, and weight restoration [2,25]. Nutritional rehabilitation may be complemented by psychological support and multidisciplinary care. However, menstrual recovery may be delayed or incomplete despite lifestyle intervention, supporting consideration of additional treatment in selected patients [23].

Hormonal therapy may be considered when menstrual function and bone health fail to improve adequately after non-pharmacological intervention. A network meta-analysis of 13 randomized controlled trials involving 897 participants found that transdermal estrogen improved lumbar spine BMD (SMD 0.34; 95% CI, 0.03–0.64) and femoral neck BMD (SMD 0.57; 95% CI, 0.04–1.10), whereas oral hormone therapy and combined oral contraceptives (COCs) did not demonstrate significant benefits [23,26,27]. Individual randomized trials similarly demonstrated improvements in lumbar spine and femoral neck Z-scores with transdermal 17β-estradiol combined with cyclic progesterone in oligoamenorrheic athletes and improvements in lumbar spine and hip Z-scores in adolescents with AN [28,29]. The greater skeletal benefit of transdermal estrogen may partly reflect avoidance of first-pass hepatic metabolism and its suppressive effects on IGF-1 [30]. Current guidelines therefore advise against prescribing COCs solely to improve BMD and recommend considering transdermal estradiol with cyclic progestin in women with persistent amenorrhea despite an adequate trial of non-pharmacological intervention, although the certainty of evidence remains limited [1,23].

Evidence for other pharmacological interventions remains limited. Teriparatide has shown improvements in lumbar spine BMD, particularly in women with AN, but its use should be restricted to selected patients with severe skeletal impairment [23]. Bisphosphonates and denosumab are not routinely recommended in women with FHA because of limited evidence and reproductive safety concerns [23]. Pregnancy should be avoided during treatment and for at least five months after the last dose [1,23].

Follow-up and repeat DXA assessment should be individualized according to clinical course, recovery of energy availability, and additional skeletal risk factors [1,23]. BMD deficits may persist despite restoration of menstrual function, supporting continued assessment of skeletal health in women at increased risk.

## 5. Discussion

The present systematic review and meta-analysis demonstrates that women with FHA and related hypothalamic amenorrhea phenotypes have impaired skeletal health compared with healthy eumenorrheic controls. Across the eleven studies included in the systematic review, lower BMD was consistently reported in women with FHA or AN, while the magnitude and skeletal distribution of these deficits varied according to the clinical population and skeletal site. Among athletes with amenorrhea, reductions were more consistently observed at the lumbar spine, whereas hip BMD was relatively preserved in some studies, suggesting a potential modifying effect of mechanical loading on site-specific skeletal involvement. Five studies comprising 14 pairwise comparisons were included in the quantitative synthesis. Among women with FHA, BMD was reduced at the proximal hip (Hedges’ g = −0.60; 95% CI −1.13 to −0.06), while a moderate reduction was also observed at the lumbar spine (Hedges’ g = −0.68; 95% CI −1.48 to 0.13). The statistical uncertainty of the lumbar spine estimate should not be interpreted as evidence of the absence of a clinically relevant BMD deficit. These findings should be interpreted cautiously given the limited number of studies, substantial between-study heterogeneity, and sensitivity of some estimates to the statistical approach and exclusion of individual studies. In contrast, women with AN demonstrated markedly greater reductions in BMD at both the lumbar spine (Hedges’ g = −1.66; 95% CI, −2.28 to −1.04) and proximal hip (Hedges’ g = −1.37; 95% CI, −2.33 to −0.42), with the lumbar spine finding remaining statistically significant across the sensitivity analyses. These findings are consistent with current evidence indicating that impaired bone health in FHA is determined not only by hypoestrogenism but also by the severity of energy deficiency and alterations in IGF-1, leptin, cortisol, and other metabolic regulators that directly influence bone remodeling [31,32].

The leave-one-out analyses provided additional insight into the observed between-study heterogeneity. The reduction in lumbar spine BMD in women with AN was the most consistent finding, remaining large and statistically significant after exclusion of each individual study. In the FHA subgroup, exclusion of Notaristefano et al. resulted in a more pronounced reduction in lumbar spine BMD and eliminated the observed heterogeneity (I^2^ decreased from 83% to 0%). Similarly, exclusion of Ackerman et al., which specifically included amenorrheic athletes, resulted in a larger pooled reduction in hip BMD, while heterogeneity decreased from 63% to 39%. Differences in the characteristics of the populations included across studies, including athletic status, may contribute to the observed heterogeneity within the FHA analyses. Accordingly, the lumbar spine estimate in the FHA subgroup appears sensitive to study composition and should be interpreted cautiously. Clinical heterogeneity across FHA populations, including differences in athletic status, duration of amenorrhea, and diagnostic criteria, could not be reliably explored because of the small number of studies. Among women with AN, exclusion of Trombetti et al. resulted in larger pooled reductions in BMD at both skeletal sites, while heterogeneity decreased from 71% to 0% for the lumbar spine and from 89% to 59% for the hip. The populations included in Trombetti et al. and Lawson et al. differed in several clinical characteristics, including the presence or recovery of menses, duration of amenorrhea, and exposure to estrogen therapy, which may contribute to variation in the observed effect estimates. For hip BMD in AN, exclusion of Lawson et al. resulted in loss of statistical significance despite an essentially unchanged effect magnitude; the confidence interval widened and heterogeneity increased, reflecting greater uncertainty in the estimate based on the two remaining studies. Given the small number of studies in each analysis, the contribution of specific clinical characteristics to between-study heterogeneity could not be formally evaluated.

The substantially greater reductions in BMD observed in the analyses of women with AN compared with the FHA/HA analyses may reflect the greater severity and duration of energy deficiency and the associated neuroendocrine and metabolic disturbances characteristic of AN. In the studies contributing to the FHA/HA analyses, AN was either explicitly excluded or the populations were selected to represent non-AN causes of hypothalamic amenorrhea, including exercise-associated amenorrhea. Hypoestrogenism is an established contributor to bone loss, while reduced energy availability may additionally affect skeletal homeostasis through metabolic adaptations, including reduced IGF-1 and leptin and increased cortisol [5]. Recent clinical data also demonstrated an association between longer duration of amenorrhea and lower lumbar spine and femoral BMD in women with FHA [12].

Alterations in biochemical markers of bone turnover may provide additional insight into skeletal changes associated with FHA and AN. Studies in women with AN have reported alterations in markers of bone formation, including osteocalcin (OCN) and bone-specific alkaline phosphatase (ALP), as well as changes in osteopontin (OPN) and the osteoprotegerin (OPG)/RANKL system [33,34,35]. At the molecular level, Runx2 is a key regulator of osteoblast differentiation and is linked to the expression of osteogenic markers including ALP, OCN, and OPN. Experimental evidence also suggests that pigment epithelium-derived factor (PEDF) may influence osteogenic differentiation and mineralization through pathways involving Runx2, ALP, the OPG/RANKL system, and vascular endothelial growth factor (VEGF) [36]. Pituitary hormones may influence bone remodeling through both direct skeletal effects and interactions with peripheral endocrine pathways. However, these molecular pathways have not been sufficiently investigated specifically in women with FHA or AN, and their relevance to skeletal impairment in these conditions remains to be established [37].

These findings have important clinical implications. Although FHA is a potentially reversible condition, its occurrence during adolescence and early adulthood may disrupt the attainment of peak bone mass, thereby limiting optimal skeletal reserve and increasing the lifetime risk of stress and fragility fractures [5,7]. Recent epidemiological evidence further supports an increased fracture burden in populations affected by amenorrhea and severe energy deficiency. Among high-endurance female athletes, bone stress injuries were frequently reported, highlighting the clinical consequences of menstrual dysfunction in physically active women and the potential modifying role of mechanical loading [38]. In women with AN, fracture risk extends across multiple skeletal sites; in a large population-based cohort, the greatest relative increases were observed for hip/femur and pelvic fractures, while vertebral fracture risk was also significantly elevated [39]. More recent population-based studies have confirmed an increased long-term fracture burden in women with AN [40,41]. In contrast, in the international GLOW cohort of postmenopausal women, clinical vertebral fractures were somewhat more frequent than hip fractures, accounting for 8% and 6% of incident fractures, respectively [42]. Together, these findings indicate that fracture patterns are population- and site-specific and may not directly parallel differences in BMD across skeletal sites. The findings emphasize the importance of early recognition of menstrual dysfunction, timely assessment of bone health using DXA, and comprehensive correction of low energy availability, which represents the primary pathogenic mechanism underlying FHA. In women who fail to recover spontaneous menstrual function following adequate lifestyle intervention, current evidence further supports the use of transdermal 17β-estradiol combined with cyclic progesterone rather than combined oral contraceptives for the preservation of bone mass.

Beyond total energy availability, the nutritional composition of the diet may also influence skeletal health. In physically active women with menstrual dysfunction, current evidence emphasizes adequate intake of bone-related nutrients, particularly calcium and vitamin D, while phosphorus, magnesium, and other micronutrients also contribute to skeletal mineralization and metabolism. Vitamin C may additionally support bone formation through its role in collagen synthesis and osteoblast differentiation [43,44]. Dietary patterns may influence the overall availability of these nutrients: evidence from broader populations suggests more favorable skeletal outcomes with nutrient-dense and Mediterranean-type dietary patterns, whereas Western dietary patterns characterized by greater consumption of processed foods and refined products have been associated with less favorable bone outcomes; these associations may also differ across Asian populations [45]. However, direct comparative evidence evaluating Western, Mediterranean, or specific Asian dietary patterns in women with FHA remains limited, and their contribution to the skeletal deficits observed in this population cannot currently be established.

Several limitations of this study should be acknowledged. First, because fewer than ten studies were included in each meta-analytic comparison, publication bias was not formally assessed using funnel plots or statistical tests, as these methods have limited reliability under such circumstances. Second, substantial clinical and methodological heterogeneity existed across the included studies, including differences in athletic status, duration of amenorrhea, diagnostic criteria for FHA, age, DXA equipment, and methods of reporting BMD outcomes, resulting in moderate-to-high between-study heterogeneity. Given the small number of studies, these sources of heterogeneity could not be reliably explored by meta-regression. Accordingly, the pooled estimates should be regarded as preliminary and interpreted cautiously. Third, most included studies were cross-sectional, limiting the ability to infer causal relationships or evaluate long-term fracture risk. Fourth, the review protocol was not prospectively registered in PROSPERO, which limits transparency regarding the prespecification of the review methods, and the search was restricted to English-language publications, potentially resulting in the omission of relevant studies. Although most studies included in the meta-analysis were rated as having good methodological quality according to the Newcastle–Ottawa Scale, limitations in control selection and group comparability in some studies, together with clinical heterogeneity among study populations and designs, should be considered when interpreting the pooled estimates.

Despite these limitations, the present review synthesizes the most up-to-date evidence on skeletal health in women with FHA by integrating a systematic review, quantitative meta-analysis, and current clinical recommendations. The findings provide a more comprehensive assessment of the magnitude of bone loss across different clinical phenotypes of FHA and may help inform future strategies for diagnosis, monitoring, and management of affected patients.

## 6. Conclusions

Women with functional hypothalamic amenorrhea generally showed lower bone mineral density than healthy controls, although the magnitude and statistical certainty of the differences varied across skeletal sites and populations. Women included in the FHA analyses showed moderate reductions in BMD, whereas substantially greater reductions at both the lumbar spine and hip were observed in the AN analyses. These findings underscore the importance of early assessment of bone health and timely restoration of energy availability, body weight, and menstrual function. The statistical significance of several pooled estimates, particularly hip BMD in both subgroups and lumbar spine BMD in FHA, was sensitive to the analytical method or exclusion of individual studies, whereas the lumbar spine reduction in AN was the most robust finding. Given the limited number of studies, substantial between-study heterogeneity, and sensitivity of some pooled estimates to the analytical approach and exclusion of individual studies, larger prospective studies using standardized diagnostic criteria and uniform methods for skeletal assessment are needed to better define long-term fracture risk and optimize therapeutic strategies for women with FHA.

## Figures and Tables

**Table 2:** Pooled Effect Estimates for Bone Mineral Density in Women with Functional Hypothalamic Amenorrhea and Anorexia Nervosa Compared with Healthy Controls.

Comparison	Studies (*k*)	Hedges’ *g* (95% CI)	*p* Value	*I* ^2^
Lumbar spine: FHA compared with controls	4	−0.68 (−1.48 to 0.13)	0.101	83%
Hip: FHA compared with controls	4	−0.60 (−1.13 to −0.06)	0.030	63%
Lumbar spine: AN compared with controls	3	−1.66 (−2.28 to −1.04)	<0.001	71%
Hip: AN compared with controls	3	−1.37 (−2.33 to −0.42)	0.005	89%

Abbreviations: AN, anorexia nervosa; CI, confidence interval; FHA, functional hypothalamic amenorrhea.

## Data Availability

No new data were created or analyzed in this study.

## Supplementary Materials

The following supporting information can be downloaded at https://www.mdpi.com/article/10.3390/medsci14060615/s1. Table S1: PRISMA 2020 Checklist. Table S2: Newcastle–Ottawa Scale Assessment of Studies Included in the Meta-analysis. Table S3: Sensitivity Analysis of Pooled BMD Estimates Using REML Estimation with the Hartung–Knapp Adjustment. Table S4: Leave-One-Out Sensitivity Analysis of Pooled BMD Estimates Using the DerSimonian–Laird Random-Effects Model.

## Abbreviations

AN	Anorexia nervosa
BMD	Bone mineral density
BMI	Body mass index
CI	Confidence interval
COCs	Combined oral contraceptives
DXA	Dual-energy X-ray absorptiometry
FHA	Functional hypothalamic amenorrhea
GnRH	Gonadotropin-releasing hormone
HR-pQCT	High-resolution peripheral quantitative computed tomography
IGF-1	Insulin-like growth factor 1
IL-6	Interleukin-6
MCP-1	Monocyte chemoattractant protein 1
RANKL	Receptor activator of nuclear factor kappa B ligand
SD	Standard deviation
SEM	Standard error of the mean
SMD	Standardized mean difference
TBS	Trabecular bone score

## References

[1] Gordon C.M., Ackerman K.E., Berga S.L., Kaplan J.R., Mastorakos G., Misra M., Murad M.H., Santoro N.F., Warren M.P. (2017). Functional Hypothalamic Amenorrhea: An Endocrine Society Clinical Practice Guideline. J. Clin. Endocrinol. Metab..

[2] Indirli R., Lanzi V., Mantovani G., Arosio M., Ferrante E. (2022). Bone Health in Functional Hypothalamic Amenorrhea: What the Endocrinologist Needs to Know. Front. Endocrinol..

[3] Meczekalski B., Katulski K., Czyzyk A., Podfigurna-Stopa A., MacIejewska-Jeske M. (2014). Functional Hypothalamic Amenorrhea and Its Influence on Women’s Health. J. Endocrinol. Investig..

[4] Cacciatore C., Cangiano B., Carbone E., Spagnoli S., Cid Ramirez M.P., Polli N., Bonomi M., Persani L. (2023). Body Weight Variation Is Not an Independent Factor in the Determination of Functional Hypothalamic Amenorrhea in Anorexia Nervosa. J. Endocrinol. Investig..

[5] Wong L., Leibner L., Vicioso C., Shah B., Ranade S.C. (2025). Functional Hypothalamic Amenorrhea in Adolescent Athletes Impairs Bone Accrual and Increases Fracture Risk. Front. Endocrinol..

[6] Behary P., Comninos A.N. (2022). Bone Perspectives in Functional Hypothalamic Amenorrhoea: An Update and Future Avenues. Front. Endocrinol..

[7] De Souza M.J., Nattiv A., Joy E., Misra M., Williams N.I., Mallinson R.J., Gibbs J.C., Olmsted M., Goolsby M., Matheson G. (2014). 2014 Female Athlete Triad Coalition Consensus Statement on Treatment and Return to Play of the Female Athlete Triad: 1st International Conference Held in San Francisco, California, May 2012 and 2nd International Conference Held in Indianapolis, Indiana, …. Br. J. Sports Med..

[8] Vescovi J.D., Jamal S.A., De Souza M.J. (2008). Strategies to Reverse Bone Loss in Women with Functional Hypothalamic Amenorrhea: A Systematic Review of the Literature. Osteoporos. Int..

[9] Biller B., Coughlin J.F., Saxe V.C., Schoenfeld D., Spratt D., Klibanski A. (1991). Osteopenia in Women With Hypothalamic Amenorrhea: A Prospective Study. Obstet. Gynecol..

[10] Safwan N., Uddenberg E.R., Hurtado M.D., Saadedine M., Faubion S.S., Obrutu O., Berga S.L., Pisarska M.D., Merz C.B., Shufelt C.L. (2024). 7590 Bone Mineral Content In Women With Functional Hypothalamic Amenorrhea Compared To Eumenorrheic Controls And Recently Menopausal Women. J. Endocr. Soc..

[11] Lu Y., Lu P., Lin L., Chen H., Zhang F., Li X. (2024). Characteristics of Bone Mineral Density in Patients with Functional Hypothalamic Amenorrhoea and Its Association with Reproductive Hormones and Body Composition. Clin. Endocrinol..

[12] Safwan N., Castaneda R., Uddenberg E.R., Hurtado Andrade M.D., Maughan J., Saadedine M., Faubion S.S., Berga S.L., Pisarska M.D., Bairey Merz C.N. (2026). Bone Mineral Density in Women with Functional Hypothalamic Amenorrhea Compared to Eumenorrheic Controls and Recently Menopausal Women. Front. Endocrinol..

[13] Trombetti A., Richert L., Herrmann F.R., Chevalley T., Graf J.D., Rizzoli R. (2013). Selective Determinants of Low Bone Mineral Mass in Adult Women with Anorexia Nervosa. Int. J. Endocrinol..

[14] Ackerman K.E., Nazem T., Chapko D., Russell M., Mendes N., Taylor A.P., Bouxsein M.L., Misra M. (2011). Bone Microarchitecture Is Impaired in Adolescent Amenorrheic Athletes Compared with Eumenorrheic Athletes and Nonathletic Controls. J. Clin. Endocrinol. Metab..

[15] Grinspoon S., Miller K., Coyle C., Krempin J., Armstrong C., Pitts S., Herzog D., Klibanski A. (1999). Severity of Osteopenia in Estrogen-Deficient Women with Anorexia Nervosa and Hypothalamic Amenorrhea. J. Clin. Endocrinol. Metab..

[16] Kandemir N., Slattery M., Ackerman K.E., Tulsiani S., Bose A., Singhal V., Baskaran C., Ebrahimi S., Goldstein M., Eddy K. (2018). Bone Parameters in Anorexia Nervosa and Athletic Amenorrhea: Comparison of Two Hypothalamic Amenorrhea States. J. Clin. Endocrinol. Metab..

[17] Drinkwater B.L., Nilson K., Chesnut C.H., Bremner W.J., Shainholtz S., Southworth M.B. (1984). Bone Mineral Content of Amenorrheic and Eumenorrheic Athletes. N. Engl. J. Med..

[18] Lawson E.A., Donoho D., Miller K.K., Misra M., Meenaghan E., Lydecker J., Wexler T., Herzog D.B., Klibanski A. (2009). Hypercortisolemia Is Associated with Severity of Bone Loss and Depression in Hypothalamic Amenorrhea and Anorexia Nervosa. J. Clin. Endocrinol. Metab..

[19] Nose-Ogura S., Yoshino O., Dohi M., Kigawa M., Harada M., Kawahara T., Osuga Y., Saito S. (2020). Low Bone Mineral Density in Elite Female Athletes With a History of Secondary Amenorrhea in Their Teens. Clin. J. Sport Med..

[20] Notaristefano G., Merola A., Scarinci E., Ubaldi N., Ranalli M., Tropea A., Diterlizzi A., Fabozzi S.M., Alesiani O., Silvestrini A. (2022). Circulating Irisin Levels in Functional Hypothalamic Amenorrhea: A New Bone Damage Index? A Pilot Study. Endocrine.

[21] Haenggi W., Casez J.P., Birkhaeuser M.H., Lippuner K., Jaeger P. (1994). Bone Mineral Density in Young Women with Long-Standing Amenorrhea: Limited Effect of Hormone Replacement Therapy with Ethinylestradiol and Desogestrel. Osteoporos. Int..

[22] Davies M.C., Hall M.L., Jacobs H.S. (1990). Bone Mineral Loss in Young Women with Amenorrhoea. BMJ.

[23] Efthymiadis A., Tsikopoulos K., Mills E.G., Milne A., Dhillo W.S., Abbara A., Comninos A.N. (2026). Pharmacological Interventions to Improve Bone Density in Functional Hypothalamic Amenorrhea: A Systematic Review and Network Meta-Analysis of Randomized Clinical Trials. J. Clin. Endocrinol. Metab..

[24] Shuhart C., Cheung A., Gill R., Gani L., Goel H., Szalat A. (2024). Executive Summary of the 2023 Adult Position Development Conference of the International Society for Clinical Densitometry: DXA Reporting, Follow-up BMD Testing and Trabecular Bone Score Application and Reporting. J. Clin. Densitom..

[25] Mountjoy M., Ackerman K.E., Bailey D.M., Burke L.M., Constantini N., Hackney A.C., Heikura I.A., Melin A., Pensgaard A.M., Stellingwerff T. (2023). 2023 International Olympic Committee’s (IOC) Consensus Statement on Relative Energy Deficiency in Sport (REDs). Br. J. Sports Med..

[26] Aalberg K., Stavem K., Norheim F., Russell M.B., Chaibi A. (2021). Effect of Oral and Transdermal Oestrogen Therapy on Bone Mineral Density in Functional Hypothalamic Amenorrhoea: A Systematic Review and Meta-Analysis. BMJ Open Sport Exerc. Med..

[27] Strokosch G.R., Friedman A.J., Wu S.C., Kamin M. (2006). Effects of an Oral Contraceptive (Norgestimate/Ethinyl Estradiol) on Bone Mineral Density in Adolescent Females with Anorexia Nervosa: A Double-Blind, Placebo-Controlled Study. J. Adolesc. Health.

[28] Ackerman K.E., Singhal V., Baskaran C., Slattery M., Campoverde Reyes K.J., Toth A., Eddy K.T., Bouxsein M.L., Lee H., Klibanski A. (2019). Oestrogen Replacement Improves Bone Mineral Density in Oligo-Amenorrhoeic Athletes: A Randomised Clinical Trial. Br. J. Sports Med..

[29] Misra M., Katzman D., Miller K.K., Mendes N., Snelgrove D., Russell M., Goldstein M.A., Ebrahimi S., Clauss L., Weigel T. (2011). Physiologic Estrogen Replacement Increases Bone Density in Adolescent Girls with Anorexia Nervosa. J. Bone Miner. Res..

[30] Singhal V., Ackerman K.E., Bose A., Flores L.P.T., Lee H., Misra M. (2019). Impact of Route of Estrogen Administration on Bone Turnover Markers in Oligoamenorrheic Athletes and Its Mediators. J. Clin. Endocrinol. Metab..

[31] De Laet C., Kanis J.A., Odén A., Johanson H., Johnell O., Delmas P., Eisman J.A., Kroger H., Fujiwara S., Garnero P. (2005). Body Mass Index as a Predictor of Fracture Risk: A Meta-Analysis. Osteoporos. Int..

[32] Sowińska-Przepiera E., Krzyścin M., Syrenicz I., Orlińska A., Ćwiertnia A., Przepiera A., Jezierska K., Cymbaluk-Płoska A., Bumbulienė Ž., Syrenicz A. (2024). The Role of Glucose, Insulin and Body Fat in Assessment of Bone Mineral Density and Trabecular Bone Score in Women with Functional Hypothalamic Amenorrhea. J. Clin. Med..

[33] Viapiana O., Gatti D., Dalle Grave R., Todesco T., Rossini M., Braga V., Idolazzi L., Fracassi E., Adami S. (2007). Marked Increases in Bone Mineral Density and Biochemical Markers of Bone Turnover in Patients with Anorexia Nervosa Gaining Weight. Bone.

[34] Ostrowska Z., Ziora K., Oświȩcimska J., Świȩtochowska E., Wołkowska-Pokrywa K. (2012). Dehydroepiandrosterone Sulfate, Osteoprotegerin and Its Soluble Ligand SRANKL and Bone Metabolism in Girls with Anorexia Nervosa. Postep. Hig. Med. Dosw..

[35] Gibson D., Filan Z., Westmoreland P., Mehler P.S. (2024). Loss of Bone Density in Patients with Anorexia Nervosa Food That Alone Will Not Cure. Nutrients.

[36] Chanpaisaeng K., Dass C.R. (2025). The Role of PEDF in the Eye, Bone, and Nervous and Immune Systems. Pharmaceutics.

[37] Niwczyk O., Grymowicz M., Szczęsnowicz A., Hajbos M., Kostrzak A., Budzik M., Maciejewska-Jeske M., Bala G., Smolarczyk R., Męczekalski B. (2023). Bones and Hormones: Interaction between Hormones of the Hypothalamus, Pituitary, Adipose Tissue and Bone. Int. J. Mol. Sci..

[38] García-Alonso M., Corral-Gudino L. (2024). High Prevalence of Stress Fractures and Long-Term Amenorrhoea in High Endurance Female Athletes: The Misleading Lack of Correlation with Bone Mineral Density. J. Orthop..

[39] Nagata J.M., Golden N.H., Leonard M.B., Copelovitch L., Denburg M.R. (2017). Assessment of Sex Differences in Fracture Risk Among Patients with Anorexia Nervosa: A Population-Based Cohort Study Using the Health Improvement Network. J. Bone Miner. Res..

[40] Søeby M., Gribsholt S.B., Clausen L., Richelsen B. (2023). Fracture Risk in Patients with Anorexia Nervosa Over a 40-Year Period. J. Bone Miner. Res..

[41] Cyrenne-Dussault M., Côté-Corriveau G., Israël M., Steiger H., Chadi N., Low N., Brousseau É., Auger N. (2026). Anorexia Nervosa and Risk of Fractures: A Matched Cohort Study of 80,000 Men and Women. Am. J. Med..

[42] Costa A.G., Wyman A., Siris E.S., Watts N.B., Silverman S., Saag K.G., Roux C., Rossini M., Pfeilschifter J., Nieves J.W. (2013). When, Where and How Osteoporosis-Associated Fractures Occur: An Analysis from the Global Longitudinal Study of Osteoporosis in Women (GLOW). PLoS ONE.

[43] Rondanelli M., Peroni G., Fossari F., Vecchio V., Faliva M.A., Naso M., Perna S., Di Paolo E., Riva A., Petrangolini G. (2021). Evidence of a Positive Link between Consumption and Supplementation of Ascorbic Acid and Bone Mineral Density. Nutrients.

[44] Williams N.I., De Souza M.J., Misra M., Nattiv A., Joy E., Barrack M., Ricker E.A., Gorrell S., Koltun K.J., O’Donnell E. (2025). 2025 Update to the Female Athlete Triad Coalition Consensus Statement Part 2: Clinical Guidelines for Screening, Diagnosis, Treatment, and Return to Play for Adolescents and Adults. Sports Med..

[45] Perna S., Avanzato I., Nichetti M., D’Antona G., Negro M., Rondanelli M. (2017). Association between Dietary Patterns of Meat and Fish Consumption with Bone Mineral Density or Fracture Risk: A Systematic Literature. Nutrients.

