# Supplementary material for: Bone Mineral Density in Women with Functional Hypothalamic Amenorrhea: A Systematic Review and Meta-Analysis
Source: Med Sci (Basel). 2026 Sep 29;14(6):615. doi: 10.3390/medsci14060615 (PMC13706801; doi:10.3390/medsci14060615)
Supplement: Supplementary file 1 [file medsci-14-00615-s001.zip › medsci-4497812-supplementary.pdf]

Supplementary File

Supplementary Table S1. PRISMA 2020 Checklist

Section and Topic	Item	Checklist item	Location where item is reported
TITLE	1	Identify the report as a systematic review.	Title (p. 1)
ABSTRACT	2	See the PRISMA 2020 for Abstracts checklist.	Abstract (p. 2)
INTRODUCTION	3	Describe the rationale for the review in the context of existing knowledge.	Introduction (pp. 2–3)
	4	Provide an explicit statement of the objective(s) or question(s).	Introduction (p. 3)
METHODS	5	Specify the inclusion and exclusion criteria and how studies were grouped.	Section 2.2 (p. 4)
	6	Specify all information sources and the date when each source was last searched.	Section 2.2 (p. 4)
	7	Present the full search strategies for all databases.	Section 2.2 (search terms and databases described) (p. 4)
	8	Specify the methods used to determine study eligibility.	Section 2.3 (p. 4)
	9	Specify the methods used to collect data from reports.	Section 2.3 (p. 4)
	10a	List and define all outcomes for which data were sought.	Sections 2.3–2.4 (pp. 4–5)
	10b	List and define all other variables for which data were sought.	Section 2.3 (p. 4)
	11	Specify the methods used to assess risk of bias.	Section 2.4 (Newcastle–Ottawa Scale) (p. 5)
	12	Specify the effect measure(s).	Section 2.4 (p. 5)
	13a	Describe the processes used to decide study eligibility for synthesis.	Section 2.4 (p. 5)
	13b	Describe data preparation methods.	Section 2.4 (p. 5)
	13c	Describe methods used to tabulate or visually display results.	Section 2.4 (p. 5)
	13d	Describe methods used to synthesize results.	Section 2.4 (p. 5)
	13e	Describe methods used to explore heterogeneity.	Section 2.4 (p. 5)
	13f	Describe sensitivity analyses.	Section 2.4 (p. 5)
	14	Describe methods used to assess reporting bias.	Not performed (p. 5)
	15	Describe methods used to assess certainty of evidence.	Not performed
RESULTS	16a	Describe the search and selection process.	Section 3.1; Figure 1 (pp. 4–5)
	16b	Cite studies excluded after full-text review.	Figure 1 (p. 5)
	17	Cite each included study and present its characteristics.	Table 1 (pp. 6–8)

	18	Present assessments of risk of bias.	Section 3.2 (Newcastle–Ottawa Scale) (p. 9)
	19	Present results of individual studies.	Table 1; Figures 2–3 (pp. 6–10)
	20a	Summarize characteristics and risk of bias among studies contributing to each synthesis.	Section 3.2 (pp. 9–10)
	20b	Present results of statistical syntheses.	Table 2; Figures 2–3 (pp. 9–10)
	20c	Present investigations of heterogeneity.	Section 3.2 (p. 9)
	20d	Present sensitivity analyses.	Section 3.2; Discussion; Supplementary Table S3–S4
	21	Present assessments of reporting bias.	Not assessed
	22	Present assessments of certainty of evidence.	Not assessed
DISCUSSION	23a	Provide a general interpretation of the results.	Discussion (pp. 13–14)
	23b	Discuss limitations of the included evidence.	Discussion (p. 14)
	23c	Discuss limitations of the review processes.	Discussion (p. 14)
	23d	Discuss implications for practice and future research.	Discussion and Conclusions (pp. 13–14)
OTHER INFORMATION	24a	Provide registration information.	Section 2.2 (p. 4)
	24b	Indicate where the review protocol can be accessed.	Not applicable
	24c	Describe amendments to protocol.	Not applicable
	25	Describe sources of financial or non-financial support.	Funding (p. 14)
	26	Declare competing interests.	Conflicts of Interest (p. 14)
	27	Report availability of data, code and other materials.	Supplementary Materials (Supplementary File S1) (p. 15)

Supplementary Table S2. Newcastle–Ottawa Scale Assessment of Studies Included in the Meta-analysis

Newcastle–Ottawa Scale criterion	Grinspoon et al., 1999	Ackerman et al., 2011	Notaristefano et al., 2022	Lawson et al., 2009	Trombetti et al., 2013
**Selection**					
Adequate case definition	★	★	★	★	★
Representativeness of the cases	★	★	–	★	★
Selection of controls	★	★	★	★	–
Definition of controls	★	★	★	★	★
**Comparability**					
Comparability of cases and controls on the basis of design or analysis	★★	★★	★★	★	★
**Exposure**					

Ascertainment of exposure	★	★	★	★	★
Same method of ascertainment for cases and controls	★	★	★	★	★
Non-response rate	—	—	—	—	—
Total score	8/9	8/9	7/9	7/9	6/9

Note: ★ indicates that the Newcastle–Ottawa Scale (NOS) criterion was met; — indicates that no star was awarded. The NOS assesses three domains: selection of study groups (maximum 4 stars), comparability of groups (maximum 2 stars), and ascertainment of exposure (maximum 3 stars), with a maximum total score of 9 stars. For control selection, a star was awarded when controls were considered to originate from the same source population as cases. Notaristefano et al. (2022) recruited controls from the same outpatient setting as cases, whereas Trombetti et al. (2013) recruited controls separately, mainly among hospital staff and their families.

Supplementary Table S3. Sensitivity analysis of pooled BMD estimates using REML estimation with the Hartung–Knapp adjustment

Outcome	k	Primary DL analysis, Hedges' g (95% CI)	REML + Knapp–Hartung, Hedges' g (95% CI)	p value (REML + KH)
FHA — lumbar spine	4	−0.68 (−1.48 to 0.13)	−0.67 (−1.89 to 0.54)	0.176
FHA — hip	4	−0.60 (−1.13 to −0.06)	−0.60 (−1.50 to 0.31)	0.127
AN — lumbar spine	3	−1.66 (−2.28 to −1.04)	−1.65 (−2.87 to −0.43)	0.028
AN — hip	3	−1.37 (−2.33 to −0.42)	−1.37 (−3.29 to 0.55)	0.092

Abbreviations: AN, anorexia nervosa; BMD, bone mineral density; CI, confidence interval; DL, DerSimonian–Laird; FHA, functional hypothalamic amenorrhea; KH, Knapp–Hartung; REML, restricted maximum likelihood.

Supplementary Table S4. Leave-One-Out Sensitivity Analysis of Pooled BMD Estimates Using the DerSimonian–Laird Random-Effects Model

Outcome	Study omitted	Hedges' g	95% CI	p value	I^2 (%)
FHA — lumbar spine	Notaristefano et al., 2022	−1.071	−1.473 to −0.668	<0.001	0.0
	Grinspoon et al., 1999	−0.513	−1.524 to 0.498	0.320	84.7
	Lawson et al., 2009	−0.516	−1.515 to 0.483	0.312	86.3
	Ackerman et al., 2011	−0.635	−1.728 to 0.457	0.254	88.3
FHA — hip	Notaristefano et al., 2022	−0.676	−1.434 to 0.083	0.081	72.7
	Grinspoon et al., 1999	−0.463	−1.135 to 0.209	0.177	66.5
	Lawson et al., 2009	−0.432	−1.020 to 0.157	0.151	62.2
	Ackerman et al., 2011	−0.799	−1.269 to −0.329	<0.001	38.5
AN — lumbar spine	Grinspoon et al., 1999	−1.447	−2.106 to −0.788	<0.001	60.7
	Trombetti et al., 2013	−1.994	−2.483 to −1.505	<0.001	0.0
	Lawson et al., 2009	−1.590	−2.457 to −0.723	<0.001	82.2
AN — hip	Grinspoon et al., 1999	−0.956	−1.688 to −0.225	0.010	71.6
	Trombetti et al., 2013	−1.797	−2.544 to −1.050	<0.001	58.9
	Lawson et al., 2009	−1.378	−2.867 to 0.112	0.070	93.9

Note: Each study was sequentially omitted and the pooled effect estimate recalculated using the DerSimonian–Laird random-effects model. Negative Hedges' g values indicate lower BMD in women with FHA or AN compared with healthy controls.

Abbreviations: AN, anorexia nervosa; BMD, bone mineral density; CI, confidence interval; DL, DerSimonian–Laird; FHA, functional hypothalamic amenorrhea.
